# Novel Nano-composites SDC–LiNaSO_4_ as Functional Layer for ITSOFC

**DOI:** 10.1007/s40820-015-0038-4

**Published:** 2015-04-04

**Authors:** Weiming Lv, Ze Tong, Yi-Mei Yin, Jiewei Yin, Zi-Feng Ma

**Affiliations:** 1 Shanghai Electrochemical Energy Devices Research Center, School of Chemistry and Chemical Engineering, Shanghai Jiao Tong University, Shanghai, 200240 People’s Republic of China; 2 Shanghai Research and Development Center for Polymer Materials, Shanghai, 200235 People’s Republic of China

**Keywords:** Nano-composite, SDC–LiNaSO_4_, Ionic conductor, Solid oxide fuel cell

## Abstract

As an ionic conductive functional layer of intermediate temperature solid oxide fuel cells (ITSOFC), samarium-doped ceria (SDC)–LiNaSO_4_ nano-composites were synthesized by a sol–gel method and their properties were investigated. It was found that the content of LiNaSO_4_ strongly affected the crystal phase, defect concentration, and conductivity of the composites. When the content of LiNaSO_4_ was 20 wt%, the highest conductivity of the composite was found to be, respectively, 0.22, 0.26, and 0.35 S cm^−1^ at temperatures of 550, 600, and 700 °C, which are much higher than those of SDC. The peak power density of the single cell using this composite as an interlayer was improved to, respectively, 0.23, 0.39, and 0.88 W cm^−2^ at 500, 600, and 700 °C comparing with that of the SDC-based cell. Further, the SDC–LiNaSO_4_(20 wt%)-based cell also displayed better thermal stability according to the performance measurements at 560 °C for 50 h. These results reveal that SDC–LiNaSO_4_ composite may be a potential good candidate as interlayer for ITSOFC due to its high ionic conductivity and thermal stability.

## Introduction

Solid-state ionic conductors and their applications as electrolytes and functional interlayers in intermediate temperature solid-state fuel cells (ITSOFC) have attracted much attention in the past few years [1–5]. This is because ionic conduction in either electrolyte or electrode is crucial to good performance of a fuel cell [6–8], and it is strongly desired to look for new ionic (oxide ion and proton) conductors operating at low and intermediate temperatures [9, 10]. The use of ceria-based materials, especially samaria- or gadolinia-doped ceria (SDC or GDC) as electrolyte or as functional layers between electrolyte and electrode, can significantly lower the operation temperature and increase the performance of solid oxide fuel cells due to their higher oxide ion conductivity (0.02 S cm^−1^ at 700 °C) [11, 12]. The reason is that the ceria-based interlayer can reduce ohmic resistance and electrode polarization by increasing the electrical and ionic conduction at interface, blocking the inter-diffusion of elements and decreasing the thermal mismatch between electrode and electrolyte [3, 13]. Therefore, the enhanced ionic conductivity of the interlayer is important to improve the performance of a cell. It has been turned out that a very efficient method of enhancing the conductivity of ionic conductors is to decorate the grain boundaries with surface active second-phase particles [14]. Zhu et al. [15], Gao et al. [16], and Xia et al. [17] reported that composite electrolytes made of SDC and carbonates can promote the conductivity of SDC for one order of magnitude at 400–500 °C. They also proposed the mechanism for carbonates improving the ion conductivity of the composite electrolytes. When the temperature reaches the melting point of the second phase salt, the molten salt fills in the voids between SDC particles and thus greatly increases the concentration of defects. This will increase the interfacial area between salts and SDC and also significantly accelerates the ion conduction velocity in the grain boundary. However, the molten phase of the carbonate salts is vulnerable under long-time heating by losing mass as operating at low and intermediate temperatures (400–700 °C), and then results in instability of a cell.

In this paper, we choose alkali double sulfate LiNaSO_4_ as the second phase to prepare SDC–LiNaSO_4_ (0–30 wt%) composites because LiNaSO_4_ shows superionic conducting property at 514–615 °C [18]. We suppose that SDC–LiNaSO_4_ composites may display enhanced conductivity and higher stability than SDC–carbonate composites in intermediate temperature. The physical–chemical properties including conductivity of SDC–sulfate composites were characterized. Power densities of the cells using pure SDC and SDC–LiNaSO_4_ (20 wt%) as interlayers between yttria-stabilized zirconia (YSZ) electrolyte and Pr_0.5_Sr_0.5_Fe_0.8_Cu_0.2_O_3−δ_ (PSFC) [19] cathode were also compared.

## Experimental

### Preparation of SDC (Ce_0.8_Sm_0.2_O_1.90_) and SDC–LiNaSO_4_

A sol–gel method was used to synthesize SDC. The typical process is that cerium nitrate and samarium nitrate were dissolved into deionized water with a molar ratio of *n*_Ce3+_: *n*_Sm3+_ = 4:1. Then EDTA and citric acid were added in the solution with the total molar ratio of *n*_*M*_:*n*_EDTA_:*n*_CA_ = 1:1:1.2. The pH of the solution was adjusted to pH 7–8 by adding ammonia. After that, the solution was heated and stirred at about 85 °C until it turned into a transparent gel. The gel was dried at 250 °C for 5 h and then a solid precursor was obtained. The precursor was finally calcined at 500 °C for 5 h to produce the SDC powder.

LiNaSO_4_ was prepared by mixing Li_2_SO_4_·H_2_O and Na_2_SO_4_ with a molar ratio of 1:1, ball-milling for 2 h, and calcining at 600 °C for half an hour. The as-prepared SDC and LiNaSO_4_ powders were mixed with proper mass ratio (0–30 wt% for LiNaSO_4_ powder) and pre-calcined at 750 °C for 1 h to obtain SDC–LiNaSO_4_ composites. For X-ray diffraction (XRD) measurements, the LiNaSO_4_, SDC, and SDC–LiNaSO_4_ composite powders were pressed to wafers under 100 MPa and sintered at 950 °C for 0.5 h. The composites and pure SDC were sintered, respectively, at 1050 and 1300 °C for 5 h for conductivity measurements.

### Characterization

The compatibility of SDC and LiNaSO_4_, crystal structure, size, and morphology of composites were investigated by XRD (Rigaku D/max-2200/PC) and transmission electron microscope (TEM, JEM-2010/INCA). The Fourier transform infrared spectra (FTIR) measurements were performed on Paragon 1000 (Perkin Elmer USA). The phase transition and thermal stability were investigated by a thermal gravimetric (TG) differential scanning calorimetric (DSC) techniques.

The ionic conductivity measurements were performed via AC impedance method using an electrochemical workstation (Zahner IM6eX) under open-circuit voltage (OCV) in air. A 2-probe setup with silver paint electrodes was used. The applied frequency was ranged from 0.01 Hz to 1 × 10^6^ Hz, and the signal amplitude was 50 mV. Measurements were conducted in 400–700 °C in a 25 °C interval.

Anode supported single cells were fabricated using YSZ as electrolyte with the thickness of about 18 μm. Two configurations of the cells respectively are Ni-YSZ/YSZ/SDC–LiNaSO_4_/PSFC (cell (a)) and Ni-YSZ/YSZ/SDC/PSFC (cell (b)). The interlayer was firstly screen printed onto YSZ side of Ni-YSZ/YSZ half-cell, and sintered, respectively, at 1050 and 1200 °C for 2 h for SDC–LiNaSO_4_ (20 wt%) and SDC. Then, the PSFC cathode was screen printed onto the interlayer and sintered at 950 °C for 2 h. The current–voltage curves of single cells were measured by a sourcemeter (Keithley 2420, America). The anode side of the single cell was fed by humidified hydrogen with 3 vol% H_2_O at the flow rate of 60 mL min^−1^, while the cathode was exposed to air.

## Results and Discussion

### TG–DSC Analysis of LiNaSO_4_

**Fig. 1:**
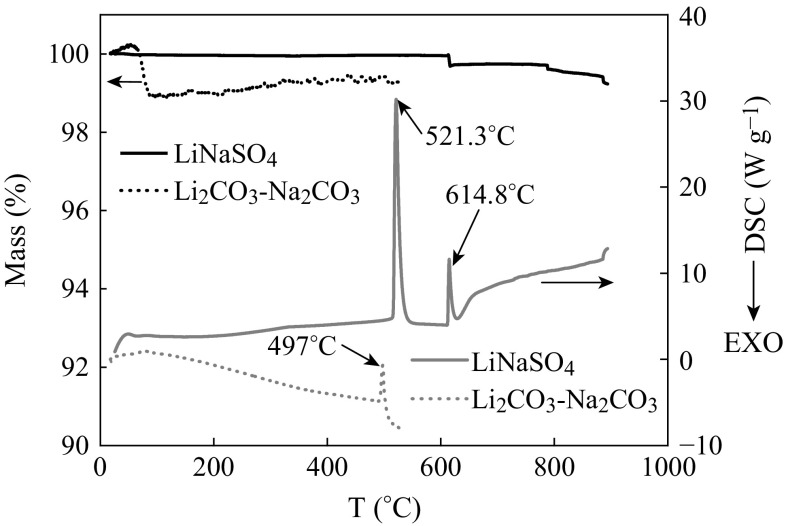
TG and DSC curves of LiNaSO_4_ and Li_2_CO_3_–Na_2_CO_3_

The TG and DSC curves of LiNaSO_4_ are shown in Fig. 1. As a comparison, the results of binary carbonate Li_2_CO_3_–Na_2_CO_3_ are also displayed. Two sharp endothermic peaks for DSC spectrum of LiNaSO_4_ in position at about 521 and 615 °C are, respectively, attributed to a phase transition from hexagonal to body-centered cubic (bcc) phase and the melting of LiNaSO_4_ [20]. The cations (Li^+^ and Na^+^) in the bcc phase have a high mobility according to a “paddlewheel mechanism”, in which the strongly coupled rotational motion of the translational static sulfate ions enhances the mobility of the cations. This is the reason that LiNaSO_4_ was reported be a superionic conductor at high temperature [18, 21, 22]. The TG curve of LiNaSO_4_ in Fig. 1 displays that the weight is almost unchanged in 60–615 °C, and only a small weight loss of 0.24 % occurred above the melting point of 615 °C. With further increase in the temperature, no weight loss occurred in 615–788 °C in the melting process of LiNaSO_4_, indicating that the material is thermally stable up to about 788 °C. Comparing with the binary salt Li_2_CO_3_–Na_2_CO_3_ (dotted lines) whose melting point is 497 °C, LiNaSO_4_ shows relative higher thermal stability in the intermediate temperature range of 500–700 °C.

### Compatibility, Phase Structure, Size, and Morphology of SDC–LiNaSO_4_

**Fig. 2:**
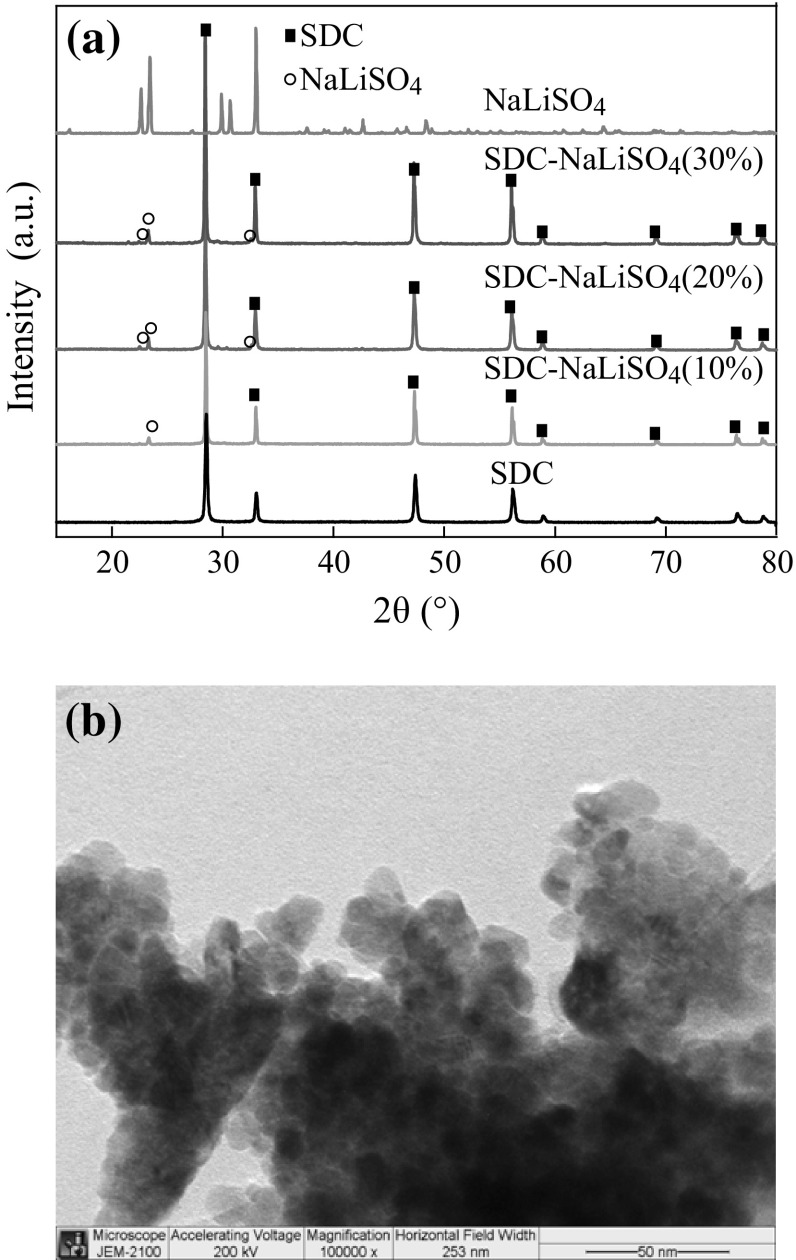
**a** XRD patterns of pure LiNaSO_4_, SDC, and SDC–LiNaSO_4_ (10–30 wt%) wafers sintered at 950 °C for 0.5 h; **b** TEM image of SDC–LiNaSO_4_ (20 wt%) powders calcined at 750 °C for 1 h

The XRD patterns of pure LiNaSO_4_, SDC, and SDC–LiNaSO_4_ (10–30 wt%) wafers are shown in Fig. 2a. The crystallite size and the volume of crystal unit cell were calculated using Scherrer’s formula and are listed in Table 1. It can be seen that no alien peaks appear except of LiNaSO_4_ and SDC, indicating that no reaction occurred between LiNaSO_4_ and SDC in the calcined and test processes. However, the intensity of SDC peaks was enhanced with the addition of LiNaSO_4_, especially for the 30 wt% sample. Contrarily, the intensity of LiNaSO_4_ peaks was significantly reduced and only the peak at about 2*θ* = 23° was identified. These results demonstrate that the crystallization of SDC was slightly promoted by LiNaSO_4_, whereas the crystallization of LiNaSO_4_ was suppressed evidently by the presence of SDC. The crystallite sizes of all samples are in nanoscale as shown in Table 1. The peak positions for both SDC and LiNaSO_4_ were found to shift to lower 2*θ* with increasing *x* as shown in Fig. 2a. This reveals the enlargement of interplanar crystal spacing and lattice expansion occurred for both constituents of the composites compared to pure SDC and LiNaSO_4_. The lattice expansion was also confirmed by the calculated volume of crystal unit cell of SDC (Table 1). This may be attributed to the formation of more defects/vacancies during the quenching process. Because of the radius difference between cations (Li^+^, Na^+^) and anion (SO_4_^2−^), LiNaSO_4_ is more likely to form Frenkel defects, i.e., M_i_· and V_M′_ (M = Li, Na), which are mainly exist in the interstitial of a crystal material. The production of interstitial defects will lead to lattice expansion. The formation of defects in the lattice of LiNaSO_4_ would cause lattice distortion, which would be expected to be reflected in the infrared spectra of the material.

**Table 1:** The crystallite size and unit cell volume of the samples in Fig. 2a

Samples	SDC	10 wt%	20 wt%	30 wt%	LiNaSO_4_
Crystallite size (nm)	47.2	58.5	60.9	79.9	69.2
Vol (Å^3^)	159.1	159.2	160.4	160.4	495.7

The representative TEM image of SDC–LiNaSO_4_ (20 wt%) powder calcined at 750 °C for 1 h is shown in Fig. 2b. It clearly shows 10–20 nm round-shaped agglomerated nanocrystallites of SDC–LiNaSO_4_ (20 wt%).

### FTIR Analysis

**Fig. 3:**
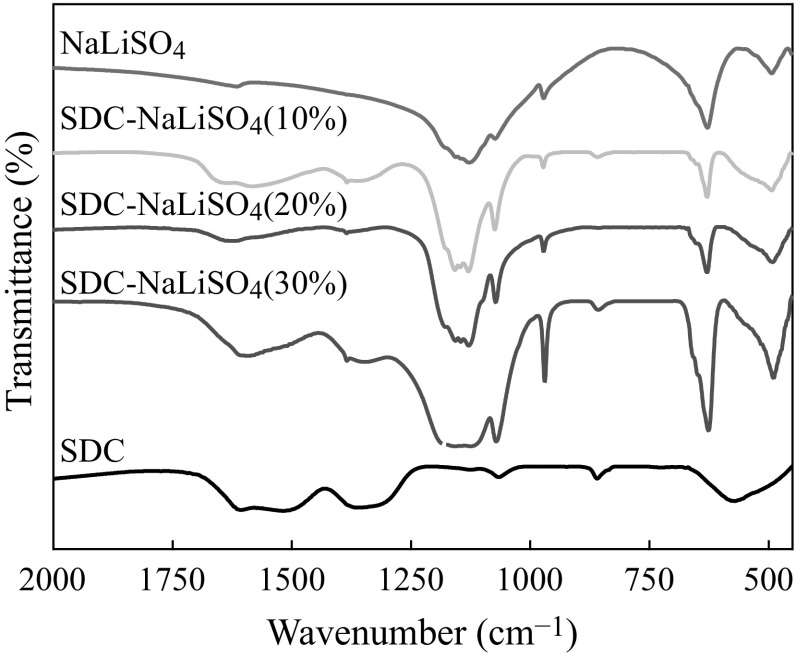
FTIR spectra for LiNaSO_4_, SDC, and SDC–LiNaSO_4_ (10–30 wt%) composites at room temperature

Figure 3 shows the FTIR spectra of pure LiNaSO_4_, SDC, and SDC–LiNaSO_4_ composites. In crystalline solids of LiNaSO_4_, the vibration, stretching, and bending of sulfate groups are influenced by the existence of defects and symmetry variations caused by structural environment change. The appearance of ν_1_ = 971 cm^−1^ and ν_2_ = 492 cm^−1^ in pure LiNaSO_4_ indicates the decrease of symmetry of SO_4_^2−^ ions due to the formation of the lattice defects [20]. As symmetry reduced further, the degeneracy in ν_2_, ν_3_, and ν_4_ may be expected to be wholly or partially removed. This is confirmed by the broadening adsorption peak of ν_3_ = 1131 cm^−1^ and the split ν_4_ adsorption at 628 and 650 cm^−1^ for composites. The triply degenerate mode ν_3_ is wholly split for composites with 10–20 wt% LiNaSO_4_ as shown in Fig. 3. Moreover, for LiNaSO_4_, a new absorption appears at ν = 1073 cm^−1^. In existence of SDC, the relative intensity of this absorption increases and the peak position shifts to a higher wavenumber value along with the appearance of another new weak absorption at ν = 1180 cm^−1^. These may result from interaction of the defects with the asymmetric stretching mode ν_3_. Therefore, IR spectra indirectly demonstrate the formation of defects (more likely Frenkel defects) in LiNaSO_4_ and the concentration of the defects increases with 10–20 wt% content. It should be noted that for 30 wt% samples all the relative intensities of ν_1_–ν_4_ absorption increase significantly. This indicates that the amplitude of motions, such as vibration, stretching, and bending of sulfate groups, is obviously enhanced due to the structural environment change induced possibly by strong interactions between SDC and LiNaSO_4_. This may hinder the rotational motion of sulfate groups, and therefore decrease the mobility of the cations (Li^+^ and Na^+^).

### Conductivity of SDC–LiNaSO_4_

Conductivities of SDC and composites were determined applying the complex plane impedance analysis. Generally, three successive arcs can be observed in the complex impedance plane plots of polycrystalline solid ionic conductors, which originated, respectively, from the bulk conduction (corresponding to arc1), grain boundary resistance (corresponding to arc2), and ion transfer resistance at the electrolyte–electrode surface (corresponding to arc3 or a tail) in sequence of decreasing frequency [23–25]. The total resistance of the solid conductor is the sum of bulk resistance and grain boundary resistance, and it can be conveniently converted to conductivity by considering the thickness (*l*) and area of the sample using the equation of *σ* = *l*/RA.

**Fig. 4:**
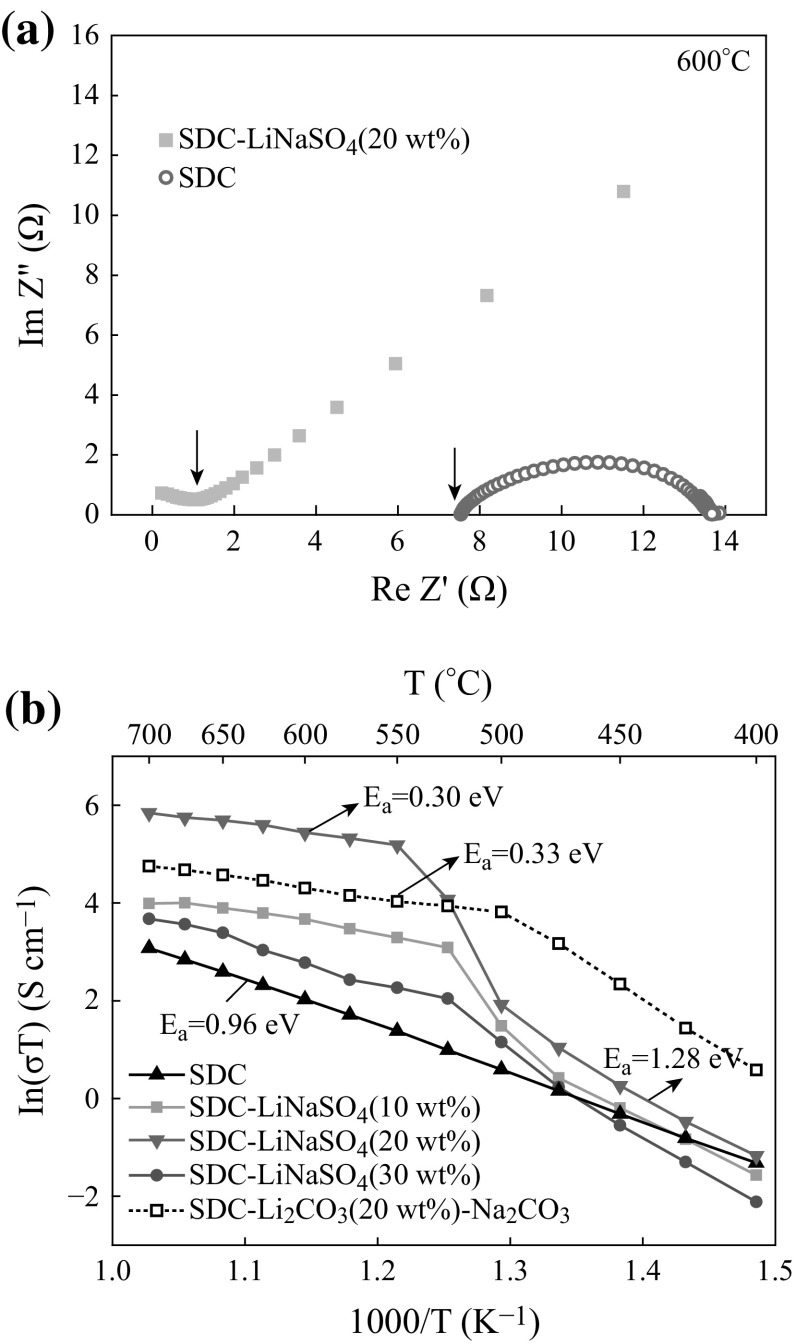
**a** Complex impedance plane plots for pure SDC and SDC–LiNaSO_4_ (20 wt%) composite at 600 °C; **b** Arrhenius plots for conductivity of SDC, SDC–LiNaSO_4_ (10–30 wt%), and SDC-(Li_2_CO_3_–Na_2_CO_3_) (20 wt%) composites

However, at a particular temperature, only parts of the three arcs appear because of the limited frequency range used in experiments. With increasing temperature, the arcs shifted to higher frequencies, leading to successive disappearance of arc1 or both arc1 and arc2. The typical complex impedance plots of pure SDC and SDC–LiNaSO_4_ (20 wt%) composite at 600 °C are shown in Fig. 4a. The total resistances are indicated by arrows in the figure. The Arrhenius plots of conductivity for SDC and SDC–LiNaSO_4_ (10–30 wt%) composites are shown in Fig. 4b. Comparably, the plot for the conductivity of SDC-(Li_2_CO_3_–Na_2_CO_3_) (20 wt%) composite is also illustrated. The conductivity of pure SDC increases linearly with temperature. From the slope, the apparent activation energy *E*_a_ = 0.96 eV was calculated, whereas the conductivities for all composites increase rapidly around the phase transition temperature of LiNaSO_4_. It can be seen that the SDC–LiNaSO_4_ (20 wt%) composite exhibits the highest conductivity at all testing temperatures compared with other SDC–LiNaSO_4_ composites. For example, it reached 0.009 and 0.217 S cm^−1^ at 500 and 550 °C, respectively. The activation energy *E*_*a*_ decreases from 1.28 eV (below the transition temperature) to 0.30 eV (above the transition temperature) which are less than those of SDC–carbonate composites (1.47 and 0.33 eV below and above the transition temperature, respectively). Moreover, the conductivity of SDC–LiNaSO_4_ (20 wt%) is much higher than that of SDC-(Li_2_CO_3_–Na_2_CO_3_) (20 wt%) at intermediate temperatures above 525 °C.

For single-phase SDC, the migration of ions is a thermally activated process. The ionic conductivity σ can be described by the Arrhenius equation of

$$ \sigma T = \sigma_{0} { \exp }\left( { - E_{\text{a}} /kT} \right), $$

where σ_0_ is a pre-exponential factor, *E*_a_ is the activation energy, *k* is the Boltzmann constant, and *T* is the absolute temperature. σ_0_ is related to two major parameters of the concentration of mobile ions (*n*) and the ion jump distance (*d*). From the equation, the decrease of σ_0_ and *E*_a_ will result in increase of the material conductivity [26]. However, some limitations exist for the conductivity enhancement of doped single-phase oxides such as SDC and YSZ: (i) high *E*_a_ values of 1.0 eV (0.96 eV in the case of SDC as shown in Fig. 4b) result from the high activation energy for the formation of oxygen vacancy, the high migration energy for transport of the oxygen ion in the confined lattice, and the strong interaction between vacancies and cations; (ii) high σ_0_ resulted from two aspects of low concentration of charge carriers due to limited doping concentration and the short jumping distance confined in a unit cell, i.e., of the order of some Angstrom.

An effective solution for above limitation is to introduce second phase to stabilize and modify the surface properties of single-phase oxides in nanoscale [27]. In nano-sized two-phase composite materials consisting of two ionic conductors, a high concentration of mobile ions, long jumping distance, and low activation energy can be obtained at the interfaces/grain boundaries without the structural limitations of the bulk. The interfacial ionic conduction mechanism allows ions to move on particles’ surface or interface through high conductivity pathways. It has been experimentally found that superionic conduction occurs for the SDC–carbonate two-phase materials, e.g., SDC–Na_2_CO_3_, SDC–CaCO_3_, SDC–Li_2_CO_3_–Na_2_CO_3_, and SDC–Li_2_CO_3_–Na_2_CO_3_–K_2_CO_3_ [16, 28–30].

Different from SDC–carbonate composites in which the significantly enhanced conductivity in 400–500 °C results from the melting process of the carbonates at the elevated temperature, the enhanced ionic conductivity of SDC–LiNaSO_4_ composites in 500–550 °C may be co-contributed by the high mobility of cations (Li^+^ and Na^+^) in bcc phase of LiNaSO_4_ at higher temperatures and the increased mobility of ions (Li^+^, Na^+^, and oxide ions) in the bulk as well as at the interface of the two constituents in the composites. The possible reasons may be as follows: (i) The discontinuity of the conductivities for the composites at 500–550 °C agrees well with the phase transition of LiNaSO_4_ (514–560 °C) instead of melting process as seen in Figs. 1 and 4; (ii) The lattice expansion and formation of more defects in the bulk of the two constituents of the composites, as analyzed in XRD and FTIR results, are beneficial to the migration of ions because ions diffuse faster in open lattice and in crystal with more defects; (iii) The bcc phase of LiNaSO_4_ is plastic crystal (rotator phase) [18], which is softer than hexagonal phase and may enhance the mobility of ions at the interface of SDC and LiNaSO_4_ at higher temperatures. The contribution of the migration of sulfate groups to the enhanced conductivity of the composites could be neglected because the diffusion coefficient of sulfate group is 4 orders of magnitude smaller compared to the monovalent cations (Li^+^ and Na^+^) [18]. In addition, proton conduction plays an important role for solid sulfate electrolytes when they are tested in hydrogen concentration cells or used as electrolyte in fuel cells [31–34]. However, in the case of this paper, for example, the conductivities were tested in air, and SDC–LiNaSO_4_ (20 wt%) was used as interlayer between YSZ electrolyte (oxide ion conductor) and cathode, the contribution of proton conduction to the conductivity of the composites should be insignificant.

When LiNaSO_4_ grains are not effectively contacted with each other just like that in SDC–LiNaSO_4_ (10 wt%), or when the rotational motion of sulfate groups in LiNaSO_4_ is hindered just like that in SDC–LiNaSO_4_ (30 wt%), the conductivity of composites will reduce. Thus, the conductivity will reach the highest value when the mass ratio of LiNaSO_4_:SDC is optimized just like that in SDC–LiNaSO_4_ (20 wt%). Moreover, the solid nature of SDC–LiNaSO_4_ composites enables them more thermally stable at intermediate temperatures of 500–600 °C.

### Single-Cell Performance with SDC and SDC–LiNaSO_4_ (20 wt%) Interlayers

**Fig. 5:**
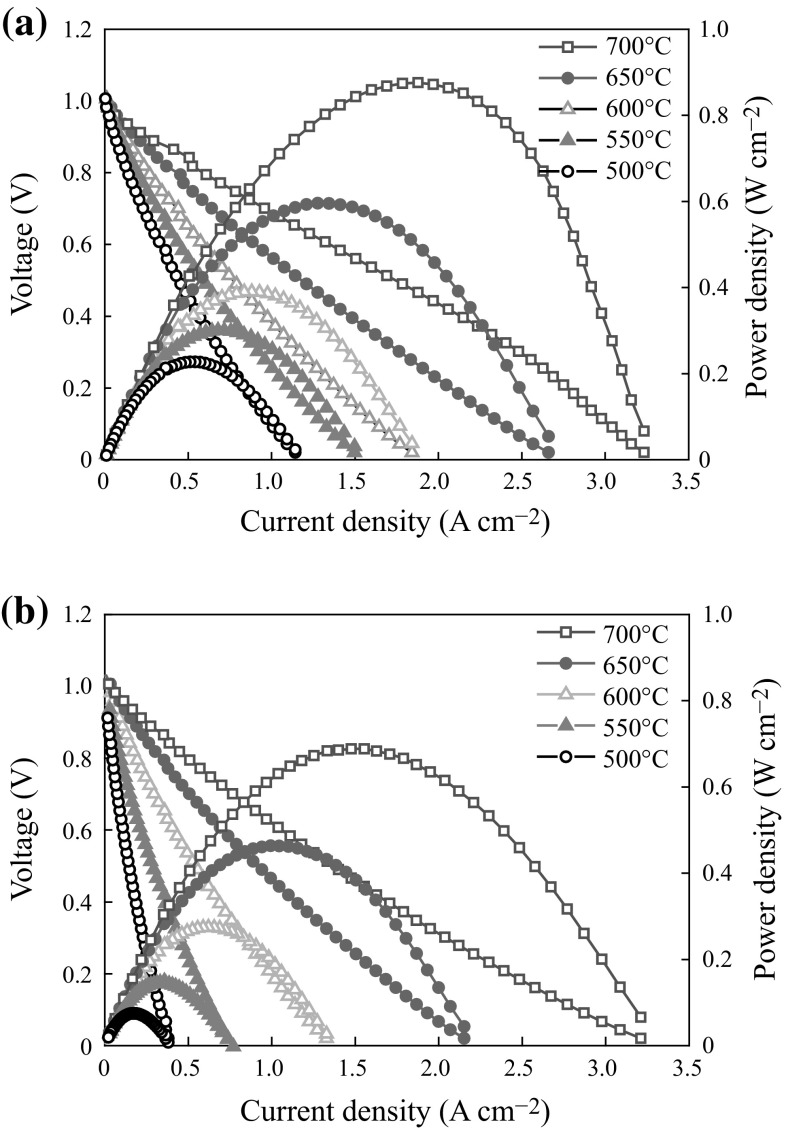
*I*–*V* and *I*–*P* curves of single cells **a** using SDC–LiNaSO_4_ (20 wt%) and **b** using SDC as interlayer

The *I*–*V* and *I*–*P* curves of single cells with SDC–LiNaSO_4_ (20 wt%) composite (cell (a)) or pure SDC (cell (b)) interlayer are shown in Fig. 5, respectively. It can be seen that the peak power density of cell (a) is significantly improved comparing with that of cell (b). For example, the peak power density of cell (a) is 0.88 W cm^−2^, while it is only 0.68 W cm^−2^ for cell (b) at 700 °C. The improvement of the performance is mainly due to the contribution of the higher conductivity of SDC–LiNaSO_4_ (20 wt%) interlayer. This indicates that SDC–LiNaSO_4_ is a promising candidate to replace the conventional SDC/GDC as the interlayer between YSZ and cathode. Much higher performance may be achieved by optimizing cathode microstructure and decreasing the thickness of YSZ electrolyte further.

**Fig. 6:**
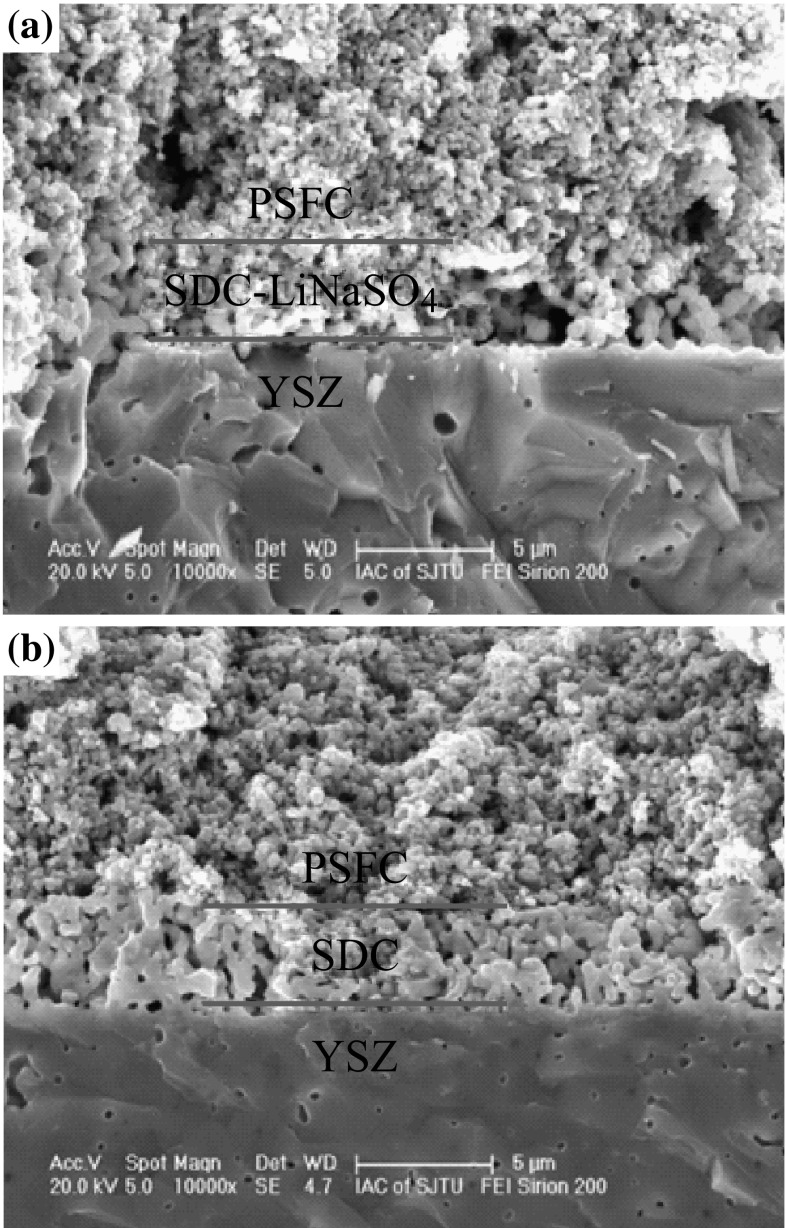
The SEM images of the cross section of single cells **a** with SDC–LiNaSO_4_ (20 wt%) interlayer; **b** with SDC interlayer

The cross-sectional SEM images of the single cells are shown in Fig. 6. One can see that three layers between the porous PSFC cathode, SDC or SDC–LiNaSO_4_ (20 wt%), and the dense YSZ electrolyte contact each other well. The thickness of the interlayer is about 3 μm. No obvious delamination or cracks were observed at interfaces, suggesting a good compatibility between these materials.

### The Stability of Single Cells

**Fig. 7:**
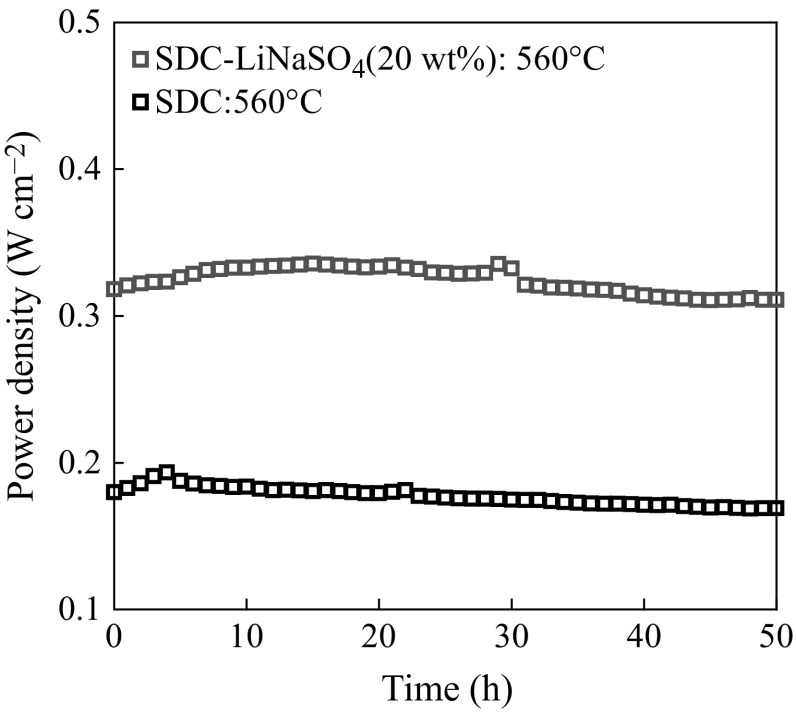
The time relationship of the power density of single cells at 560 °C for 50 h with SDC–LiNaSO_4_ (20 wt%) interlayer (*blue square*) and with SDC interlayer (*black square*). (Color figure online)

The thermal stability of SDC–LiNaSO_4_ (20 wt%) was examined by recording the performance of single cells at 560 °C for 50 h (shown in Fig. 7). The power density of both cells increases firstly and then decreases slowly. The initial improvement in performance of the cells may be attributed to re-arrangement in microstructure and activation of each constituent in the cells, whereas the subsequent decline may be due to the slight leak of gas and aggregation of cathode particles. The fluctuation of the performance of cell (a) is smaller than that of cell (b), indicating that the thermal stability of SDC–LiNaSO_4_ (20 wt%) composite is better than that of pure SDC.

## Conclusions

We prepared nano-composites SDC–LiNaSO_4_ with high ionic conductivity and thermal stability. The composites’ properties with different mass ratios were investigated and SDC–LiNaSO_4_ (20 wt%) was found to have the highest conductivity at intermediate temperature of 500–700 °C. As a functional layer of ITSOFC, the cell performance was improved significantly. Our results indicate that SDC–LiNaSO_4_ may be a penitential ionic conductive functional material for ITSOFC.
